# The hidden hurdles of clinical clerkship: unraveling the types and distribution of professionalism dilemmas among South Korean medical students

**DOI:** 10.1186/s12909-024-05115-9

**Published:** 2024-02-15

**Authors:** Ye Ji Kang, Yanyan Lin, Jaehee Rho, Jungjoon Ihm, Do-Hwan Kim

**Affiliations:** 1 Department of Medical Education, College of Medicine, Hanyang University, 222 Wangsimni-ro, Seongdong-gu, 04763 Seoul, Republic of Korea; 2 Department of Education, College of Educational Sciences, Yonsei University, 50 Yonsei-ro Seodaemun-gu, 03722 Seoul, Republic of Korea; 3 Dental Research Institute, School of Dentistry, Seoul National University, 1, Gwanak- ro, Gwanak-gu, 08826 Seoul, Republic of Korea

**Keywords:** Clerkship, Medical professionalism dilemmas, Medical students, Nun-chi, Student-doctor

## Abstract

**Background:**

To improve the medical professionalism of medical students, it is essential to understand the dilemmas they face in various situations. This study explored the types and distribution of dilemmas Korean medical students encounter during their clinical clerkships. It then compared these with previous dilemma frameworks and identified the types and distribution of “complexity dilemmas,” wherein two dilemma themes emerge in a single clinical situation.

**Methods:**

The researchers organized and recorded a group discussion with 106 third-year medical students who had completed their clinical clerkships. These students participated in the discussion as part of an assignment, focusing on the dilemmas they encountered during their clerkships. For data analysis and visualization, the researchers employed the MAXQDA software program and utilized the template analysis method, a qualitative research methodology.

**Results:**

A total of seven dilemma themes and sixteen sub-themes were identified. The identity-related dilemma concerning student-doctors had the highest frequency. The themes “mismatch” and “Nun-chi” emerged as new additions not found in previous dilemma frameworks. The complexity dilemmas appeared in the sequence of “identity-dignity,” “identity-abuse,” and “identity-consent”.

**Conclusions:**

To navigate the unique dilemmas present within South Korea’s clinical culture, several key issues need consideration: elevating the role of student-doctors, balancing the primary emphasis of educational hospitals on delivering medical services, and understanding interpersonal strategies, such as “Nun-chi”.

**Supplementary Information:**

The online version contains supplementary material available at 10.1186/s12909-024-05115-9.

## Background

Clinical clerkship offers medical students an early exposure to real-life clinical practice scenarios, which they will commonly encounter post-graduation. This practical experience marks a crucial transition from the classroom setting to the clinical workplace, typically occurring during the third year of medical school [1]. During this phase, students are expected to set learning objectives, such as performing clinical procedures on actual patients and honing their bedside manner [2, 3]. Importantly, medical students assume the legal role of “student-doctors” in clinical practice, affording them opportunities to observe physician-patient interactions, practice clinical skills, and make decisions. This hands-on experience nurtures their confidence and fosters a sense of belonging within the medical community [4, 5].

On the other hand, the transition of medical students to clerkship also impacts their professional identity formation. Professionalism is shaped gradually over time by external contexts, instruction, and reinforcement [6]. Therefore, the continuous development of professional identity is influenced by how students interpret their experiences through their own perspectives as they narrate and reflect on their everyday experiences [7]. Hence, a well-planned formal curriculum is essential.

However, the clinical learning environment is a multifaceted domain, blending education and real-world patient care, which inherently harbors uncertainties. In this high-stakes setting, patient safety is paramount, emphasizing a hierarchical communication structure and operational efficiency, potentially leading to conflicting priorities among healthcare staff [8]. These complex situations often challenge students’ ability to make independent decisions when collaborating with diverse healthcare professionals [9].

To address these challenges and guide medical students through their clinical clerkship, it is imperative to understand why they may exhibit unprofessional behaviors, experience confusion, and respond to hierarchical pressures when encountering dilemmas [10]. Implementing a formal curriculum-based orientation program that addresses these discrepancies can empower students to navigate the complexities of dilemma situations, fostering their growth into confident, patient-centered, and empathetic future medical professionals [11–13].

However, previous research on medical professionalism dilemmas has primarily focused on Western countries, potentially overlooking the unique challenges faced by medical students in diverse cultural contexts [14]. Medical students worldwide encounter both shared and context-specific dilemmas influenced by cultural factors, such as norms, ethnicity, and gender stereotypes, which profoundly shape their clinical experiences [9, 15]. Asian cultures, in particular, often emphasize collectivism and exhibit lower degrees of individualism compared to Western counterparts [16]. In collectivist cultures, healthcare professionals may hesitate in decision-making, prioritize group interests over individual concerns, and fear actions that could jeopardize their careers [9]. Cultural nuances, like South Korea’s “Nun-chi,” which emphasizes emotional intelligence and maintaining harmony within social interactions, play pivotal roles in shaping interpersonal dynamics [17]. Similar indigenous concepts exist in other Asian countries, highlighting the importance of group harmony over individuality, underscoring the significance of understanding cultural influences on dilemmas [9, 14].

Furthermore, professional dilemmas in medical education are intricate and multifaceted, leading to disagreements among faculty and students regarding what constitutes professional behavior [18]. Medical professionalism itself is multidimensional, making it challenging to formulate a single, generalized statement [19]. Dilemmas are often interconnected [20], and multiple dilemmas can coexist within a single clinical context. While there have been efforts to address this complexity, such as the study conducted by Wang & Ho [21], the methods and procedures used remain somewhat opaque. For instance, consider a scenario where a student is refused the opportunity to interview a patient due to their student status; this situation can be interpreted as an “identity-related dilemma.” Simultaneously, if the patient declines to grant consent for the medical student’s involvement, it could also be viewed as a “consent-related dilemma.” These intricate layers of interconnected dilemmas underscore the intricate nature of professional challenges in medical education.

This study identifies and comprehends the narratives regarding the dilemmas encountered by medical students during their clinical clerkship. The study therefore considered the following research questions: (1) What are the specific dilemmas that medical students encounter during their clinical clerkships? Furthermore, how do these dilemmas align with, differ from, or expand upon the dilemma framework templates established in prior research? (2) In instances where medical students grapple with more than one dilemma concurrently during their clerkship (referred to as complex dilemmas), what are the types of dilemmas involved, and how are these complex dilemmas distributed within the clinical clerkship experiences? These research questions serve as the guiding framework for our investigation into the multifaceted world of dilemmas within medical education, shedding light on the types, distribution, and complexities of these challenges encountered by students during their clinical clerkships.

## Methods

### Study design

To explore and comprehend professionalism dilemmas encountered by students during clinical clerkships, we employed narrative inquiry [22]. As an approach to exploring experiences, narrative inquiry not only focuses on individual lives as well as the broader contexts and relationships that shape those lives. This approach has been employed to examine the dilemma experiences of medical students and resident physicians across a range of contexts [23–25]. Narrative data can be collected from various sources such as video or audio transcripts, field notes, interviews, and narrative writing [26]. For this study, we collected recorded videos of student-led open discussions in situations without direct instructor facilitation, aiming to capture lived experiences of their professionalism dilemmas.

### Participants and setting

The study includes 106 third-year medical students at Hanyang University College of Medicine (HYUCM), a private medical school in South Korea. The curriculum comprises a pre-clerkship phase for the initial 2 years and a clinical clerkship phase for the final 2 years. HYUCM’s clinical clerkship is for a duration of three semesters: from the first semester of the third year to the first semester of the fourth year. The duration of clinical clerkship in the third year is 37 weeks, starting in mid-January - out of the 37 weeks, 36 weeks is for core clerkship and one week is for elective clerkship.

**Fig. 1:**
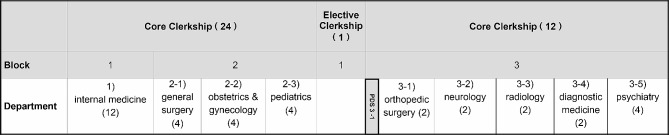
HYUCM’s clinical clerkship. The numbers in parentheses show the duration of clerkship rotations

The core clerkship consists of three blocks lasting 12 weeks each. During the first block, the focus is primarily on internal medicine. In the second block, rotations are conducted in general surgery, obstetrics and gynecology, and pediatrics. Each of these topics is covered for a period of 4 weeks each over the course of the 12-week block. The third block includes rotations in orthopedic surgery, neurology, radiology, diagnostic medicine, and psychiatry. Each topic in this block is covered for a period of 2 weeks except for psychiatry which receives 4 weeks (see Fig. 1). The cohort of approximately 110 third-year students are organized into three main groups, and within each group, there are 12 teams composed of three or four students. In other words, the 110 students are distributed among 36 teams, and these smaller teams progress through their clinical rotations in the same sequence, allowing them to have a shared experience.

Within the core clerkship, there is a one-week course called “Patient-Doctor-Society 3 − 1” (referred to as “PDS 3 − 1”). Throughout the medical curriculum, all students undertake Patent-Doctor-Society (PDS), a course series for personal and professional development. In the pre-clerkship phase, students take PDS 1, PDS 2, and PDS 3, focusing on behavioral and social sciences, with a specific emphasis on medical humanities, ethics, and professionalism. The unique aspect of PDS 3 − 1 lies in its emphasis on clinical cases encountered by students during their clerkship. The course incorporates both lectures and discussions aimed at deepening students’ understanding and application of medical professionalism. Although all 12 h of lectures along with four hours of case-based discussions were conducted online in 2021 due to the COVID-19 pandemic, it is important to note that all the students who participated in this study participated in the clinical clerkship curriculum on a face-to-face basis.

### Data collection

Data was collected in the PDS 3 − 1 course. As part of the course assignment, students worked in teams of three to four members undergoing a clinical rotation together, conducting a discussion centered on the topic “What dilemmas did you face during clerkship?”. To validate the discussion’s quality, each team was required to submit a recorded video of the entire conversation. Students were prompted to engage in discussions with their team members, sharing instances of dilemmas they had faced during their clinical clerkship. Teams were expected to hold discussions at a time and place of their preference, outside of class. There were no prescribed guidelines for how the discussion should be conducted, structured, or recorded. Students were encouraged to freely explore all aspects of professionalism, encompassing formal, informal, and hidden curricular elements. At the end of the course, all but one of the 36 teams submitted videos, resulting in a total of 35 videos collected. All submitted videos lasted for a minimum of 40 min, with a maximum duration of 50 min.

### Data analysis

The researchers followed the Standards for Reporting Qualitative Research (SRQR) to guide the analysis and reporting of findings [27]. All recorded videos were transcribed and information that could identify the students’ personal information was deleted or anonymized in order to protect the participants’ identity. Although the contents of the entire student discussion were transcribed, the researchers extracted and coded only those contents corresponding to the students’ dilemmas using the MAXQDA 2020 [28].

To analyze the data in this study, the researchers employed the template analysis method. This approach involves the application of a pre-existing template that has been used in previous research. The initial template can be modified based on the data obtained, allowing for the addition of new codes through an inductive process [29]. One notable advantage of template analysis is its capability for parallel coding, meaning that a single segment of data can be classified under multiple codes at the same level. This method offers flexibility as it allows researchers to utilize predefined themes and make adjustments by adding new codes or removing irrelevant ones [30]. In this particular study, the researchers utilized the professional dilemma framework proposed by Monrouxe and Rees [20], which noted that the themes of the dilemmas faced by students can be complex and multiple, as an initial template for analyzing their data. They conducted a large-scale international qualitative study using the narrative interviewing technique, providing medical students with a forum to recount events they perceived as professional dilemmas during their undergraduate education [10, 11, 20]. The dilemmas were categorized into six types: (1) the identity-related dilemma arising from the status of clerkship (2) [22]the abuse-related dilemma such as bullying as well as verbal and physical asymmetrical influence from hierarchies, (3) the dignity-related dilemma in dealing with patient dignity, (4) the consent-related dilemma with regards to skipping patient consent, (5) the safety-related dilemma including commissions and omissions affecting the patients’ safety, and (6) the e-professional dilemma which includes both online and Internet-based communication issues in the medical workplace [20]. This framework served as a guide during the analysis process and provided a structure for identifying and categorizing relevant themes within the collected data.

All 35 videos submitted were included in our analysis. To initiate the analysis, the first cycle of coding involved the examination of videos from 10 teams. In this phase, narratives within the students’ discussion videos were independently identified and then matched to the initial template by two researchers (YYL and JHR). The narratives were differentiated based on whether the stories described by students in the videos unfolded within the same time, place, and relationship dimensions [22]. Simply put, any difference in either time, place, or relationship dimension resulted in the narrative being treated as distinct. Following the identification of narratives, they independently utilized the initial template through parallel coding, assigning one or two codes to each narrative since no more than two codes were identified within a single narrative. Throughout this coding process, the researchers discovered new themes that had not been initially anticipated. In instances where disagreements arose during coding, the researchers engaged in discussions until a consensus was reached. Once agreement was reached on the themes and their interpretation, a refined coding template was developed. The preliminary interpretation of results was shared with the entire research team for further discussion and review of the coding process. Subsequently, YYL and JHR proceeded to analyze the remaining 20 videos. Each researcher examined 10 videos, employing the refined template as a reference for their analysis. The results were interpreted through extensive discussions within the research team. The resulting themes consisted of six themes derived from the initial template, along with an additional theme that emerged during analysis. Following this, saturation was assessed by analyzing videos from the remaining 5 teams. This ensured that enough data had been examined to confirm that new themes were unlikely to emerge. The final template was then solidified based on this comprehensive analysis.

There were various methods that were used to enhance the trustworthiness and credibility of the data analysis and interpretation. First, the richness of the interpretation was supplemented by obtaining new perspectives from the analyzed data through medical education and clinical experts. In addition, to thoroughly examine the reality of South Korea regarding the dilemmas students faced during their clerkship, related research and news articles were also reviewed separately from the secured data and referenced during the analysis process [31]. Incorporating relevant literature and research facilitated a nuanced interpretation of the emerging analysis themes, considering the cultural and contextual aspects of Korean society and medical education.

In this study, the qualitative data collected was subjected to quantification and visualization to explore data distribution and identify patterns. The inclusion of quantitative data alongside qualitative data serves a purpose: while qualitative data offer a comprehensive understanding of specific settings or individuals within certain categories, quantitative data can provide a broader perspective by revealing larger patterns. By presenting quantitative evidence, the researchers were able to support their interpretations and counterclaims that emerged from the qualitative data [32]. Through analyzing the frequency and distribution of the collected data, the researchers derived implications for interventions that require greater priority. To visualize the data in this process, MAXMaps provided by MAXQDA visual tools were utilized to graphically display the relationship of each concept [33].

### Reflexivity

The research team consisted of members with diverse backgrounds and expertise in health professions education, enabling the interpretation of findings from multiple perspectives. The first author (YJK) possesses a PhD in education with a specialization in English literature and a particular interest in student consultation and support. Both YJK and DHK were actively involved as professors in the PDS3-1 course. YYL has a medical background, having graduated from medical school in China and completed a master’s program in dermatology. JJI is engaged in the field of dental education, while JHR specializes in educational statistics and evaluation. Throughout the study, we considered the experiences and perspectives each author contributed to the research process. We engaged in reflexivity through open team dialogue and internal reflections, leveraging our diverse backgrounds to sensitively address the various dilemmas students experience. This diversity influenced our approach to analyzing and interpreting the data.

## Results

The researchers present the results in two parts:

1. What are the dilemmas faced by students during their clinical clerkship, and what are their types and distributions compared to the dilemma template used in previous studies?
2. If two or more dilemmas operate in a clerkship situation (i.e., complex dilemma), what are their types and distributions?

### Type and distribution of single dilemmas

From the 35 videos, a total of 257 narratives were identified and utilized in the final analysis. These narratives were then categorized into seven themes and 16 sub-themes, as indicated in Table 1. As parallel coding was used, the total number of codes was 293—more than the total number of narratives. Among the dilemmas encountered by medical students during their clinical clerkship, the dilemma related to “identity” ranked the highest. It was followed by “abuse,” “mismatch,” “dignity,” “consent,” “safety,” and “E-professionalism dilemma.” Compared to the dilemma framework proposed by Monrouxe and Rees [20], the main theme, “mismatch,” and the sub-theme “Nun-chi,” were newly emerging themes in this study.

**Table 1:** List of dilemma themes during clinical clerkship

Themes	Sub-themes	N	%	Complex N	Complex %
1. Identity	1–1. Being excluded from learning opportunities (from professors, nurses, patients) 1–2. Voluntarily or involuntarily concealing clerkship status (from patients)	92	35.80%	28	10.89%
2. Abuse	2 − 1. Chores unrelated to patient-care performed by students or colleagues 2–2. Unfairly scolding students or colleagues 2–3. “Nun-chi” (walking on eggshells) ^*^	65	25.29%	9	3.50%
3. Mismatch ^*^	3 − 1. Difference between knowledge and workplace 3 − 2. Difference between specialties	58	22.57%	4	1.56%
4. Dignity	4 − 1. Discrimination 4 − 2. Physical violation 4 − 3. Violation of right to know 4–4. Privacy violation	45	17.51%	18	7.00%
5. Consent	5 − 1. Conducting physical examination without the patient’s consent 5 − 2. Skipping the consent process to accomplish the task	18	7.00%	8	3.11%
6. Safety	6 − 1. Commission 6 − 2. Omission 6 − 3. Safety violation	13	5.06%	5	1.95%
7. E-professionalism	-	2	0.78%	0	0.00%
Total		293^a^	114.01%^b^	36^c^	14.01%

*Newly added theme and sub-theme

^a^A total of 257 narratives were used in the analysis, and 293 codes were derived, including those calculated in duplicate

^b^Total codes number 293

^c^As each complex dilemma story had two codes, the number of complex dilemmas = (the number of codes) 72/2

#### Identity-related dilemma

The most common dilemma experienced by medical students was related to “identity” (*n* = 92, 35.80%). Identity-related dilemma implies “being negatively affected by participation in practice as a result of the student-doctor’s status.” The theme of identity could be divided into two (2) sub-themes. The first was “being excluded from learning opportunities,” mainly by medical staff, nurses, and patients, which was caused by the failure to recognize the “existence” or legitimate affiliation of medical students (Table 2). However, in a few cases, identity-related problems caused a dilemma even in situations where participation in practice was allowed, as medical students were portrayed as students “pretending to be a doctor” voluntarily or involuntarily, in order to practice smoothly.

**Table 2:** Theme 1- the “Identity-related dilemma”

Sub-themes	Students’ dilemma narrative
Being excluded from learning opportunities (by professors, nurses, patients)	The professor tells us to actively interview the patient during practice, but I feel intimidated when the nurse scolds us, saying “Who are you?” and “What are you doing here if you are not a resident?” (Team 32) Compared to female students in obstetrics and gynecology practice, male students are often unable to participate in cesarean section and delivery. (Team 28)
Voluntarily or involuntarily concealing clerkship status (from patients)	(Involuntary) Student-doctors should always wear name tags indicating that they are students, during practice. However, in certain departments, they were told to not reveal their name tags and to answer “No” if asked whether they were students. (Team 33)
	(Voluntary) I deliberately conceal my student-doctor status because the patient might not like it that I am a student-doctor. (Team 32)

#### The abuse-related dilemma

The second type of dilemma that students experienced the most was the dilemma related to “abuse” (*n* = 65, 25.29%). This type of abuse does not refer to physical abuse, but one that is subtle in nature and an indirect form of abuse, such as an aggressive expression, a coercive atmosphere, etc., in hierarchical situations. The students expressed that they felt “shriveled” when professors “scolded” or “ignored” them; furthermore, they voluntarily restricted their behavior even without explicit coercion and “gauged others’ mood (Nun-chi)” before expressing themselves to avoid being scolded. Students also observed a similar abuse among medical personnel. Linguistic abuse mainly appeared in the form of disparaging remarks or acting in a manner that was irritating to other medical personnel. Preparation of snacks by nurses, for instance, was considered “chores unrelated to patient care” (Table 3).

**Table 3:** Theme 2– the “Abuse-related dilemma”

Sub-themes	Students’ dilemma narrative
Unfairly scolding students or colleagues	For a few professors, questions are mandatory during handover. He scolded students for not asking questions, saying, “You didn’t study!” (Team 11) We have learned to respect each other, but when visiting the operating room or outpatient clinic, the professor shows annoyance toward the nurse. (…) Professor gets angry with the nurse in the operating room. The nurses somehow hold themselves back. (Team 16)
“Nun-chi” (walking on eggshells)	In obstetrics and gynecology practice, although I had questions about textbooks or anatomical structures during surgery, I couldn’t ask because of the professor’s style and tendency, the atmosphere of the department, and the serious mood in the operating room. The resident advised me to be quiet during the surgery. I felt I had to be quiet even when I had questions. (Team 6)

#### The mismatch-related dilemma

The mismatch-related dilemma (*n* = 58, 22.57%) implies that “any differences are revalued in the context of clinical practice.” Students experienced dilemmas caused by “the difference between the principles and knowledge learned in classroom and the practices applied in the clinical workplace.” This type of dilemma (mismatch-related dilemma) is distinguished from other dilemmas in that it mainly refers to a “priority judgment between conflicting values, in consideration of the clinical context” as opposed to being a problem of “right or wrong.” An example of this in clinical decision- making situations is students witnessing physicians place more emphasis on the “cost to be charged to patients” rather than on “the treatment guidelines recommended in textbooks”. This type of dilemma also occurred with regard to interpersonal aspects such as communication or trust. An example of this is, students stated they witnessed medical staff mainly asked closed-ended questions rather than open-ended questions to make note-taking of the patients’ history shorter as well as effective under the constraint of “limited working hours.” In addition, students experienced that “trust” may not always be the top priority during the actual patient encounter, unlike what they often learned in the classroom. The students were rather advised to keep in mind that “the patient may not tell the doctor all the facts depending on the circumstances.” Finally, a few students mentioned differences in their experiences between specialties with regard to appearance, such as “necktie,” “surgical suit,” and “surgical cap” (Table 4).

**Table 4:** Theme 3 - the “Mismatch-related dilemma”

Sub-themes	Students’ dilemma narrative
Difference between knowledge and workplace	In internal medicine practice, the textbook mentioned that “antibiotics A” should be used, but the professor said, “Do not use ‘A’.” (Team 10) I was taught that when viewing medical records, only approved records should be viewed, but some professors have asked students to view even those records for which they do not have access rights, such as outpatient records. We needed the account of a resident to view these records. (Team 15)
Difference between specialties	Specialty A mentioned that neckties are likely to become contaminated and advised us against wearing them. However, Department B announced: “As you are students, wear shirt and tie with courtesy.” (Team 12)

#### The dignity-related dilemma

The dignity-related dilemma (*n* = 45, 17.51%) refers to being witness to or involved in activity that violates the dignity of the patient and is divided into four sub-themes. The sub-themes were mainly in cases of direct infringement regarding a specific patient’s dignity, such as the patient’s physical violation, right to know, and privacy. However, students experienced a high level of anguish when they felt that “patient characteristics unrelated to the disease,” such as the patient’s social status or patient–doctor intimacy, influenced clinical decisions (Table 5).

**Table 5:** Theme 4 - the “Dignity-related dilemma”

Sub-themes	Students’ dilemma narrative
Discrimination	There were two 35-week pregnant patients with identical clinical conditions. The professor recommended induction for one patient, and the other was given steroids to delay delivery. Regarding the difference, the professor said that one of the patients is an employee at this hospital, so it led to the different clinical decision. For me, it was confusing. (Team 21)
Physical violation	During the operation, a patient suffered burns because the Bovie touched the skin; there is a process of explaining such incidents to the patient’s caregiver. Although this incident occurred during the bleeding control stage, the professor explained to the caregiver that it was during the open stage. (Team 36)
Violation of the right to know	I witnessed that the resident and professor did not inform the patient about the results of the bone marrow biopsy, which was blood cancer. I thought that the patient’s right to know was violated in that the medical staff did not “break the bad news.” (Team 29)
Violation of the right to privacy	Medical staff sometimes entered the hospital room without knocking even when the door was closed or the curtains drawn. (Team 1)

#### The consent-related dilemma

The consent-related dilemma refers to the “dilemma situation related to the patient’s consent during clinical clerkship.” In this study, the frequency of students’ responses was *n* = 18 (7.00%). There were two main cases where “consent” was directly mentioned with regard to the students’ dilemma narratives—the first was the case of physical examination without consent from the supervisor. The second, was that students skipped the consent process (voluntarily) or considered incomplete consent to be sufficient to perform the tasks (assignment) that were reflected in their grades. The students’ uncertainty on whether to accomplish the tasks given or obtain consent from patients made them experience conflict within themselves (Table 6).

**Table 6:** Theme 5 - the “Consent-related dilemma”

Sub-themes	Students’ dilemma narrative
Performing physical examination without the patient’s consent	The professor gave me the opportunity to perform a DRE (digital rectal examination) in the operating room for an anaesthetized patient—without the patient’s consent. (Team 19)
Disregarding the consent process to accomplish the task	We were taught to identify ourselves as student-doctor and obtain consent. In reality, when patients lack the capacity to communicate, we often skip this step to complete the task in time. (Team 6)

#### The safety-related dilemma

The safety-related dilemma (*n* = 13, 5.06%) refers to students being a witness to or involved in errors that may affect patient safety. It was classified into three sub-themes: “Commission,” “Omission,” and “Safety violation” (Table 7). In cases of errors by “commission”, there were instances of prescribing incorrect medications or administering wrong drugs to patients. Errors by “omission” involved displaying inappropriate attitudes towards patients or delaying and neglecting crucial decisions. Lastly, “safety violations” encompass instances where hygiene-related regulations, such as handwashing or maintaining the cleanliness of medical equipment, are breached.

**Table 7:** Theme 6– the “Safety-related dilemma”

Sub-themes	Students’ dilemma narrative
Commission	The resident administered the wrong dose of antibiotics. The nursing department advised not to tell the patient about this, and the professor managed the situation by instructing the erring resident to look for reference cases related to antibiotic doses. (Team 8)
Omission	Medical personnel were not thoroughly checking patient information on the pretext that they were busy. In theory, during “history taking,” patients were taught to ask “open questions,” but in practice, they were often omitted. (Team 22)
Safety violation	A professor entered the operating room because of an emergency and wore gloves without washing his hands. (Team 18)

#### The E-professionalism-related dilemma

The least frequent dilemma was the “E-professionalism related dilemma” (*n* = 2, 0.78%), which refers to “Communicating with patients based on Social Networking Service (SNS) regardless of the practice the student was in”. Online communication with patients during the formal clerkship period was only done by a minimal number of students (Table 8).

**Table 8:** Theme 7– the “E-professionalism-related dilemma”

Sub-themes	Students’ dilemma narrative
Online communication with patients	I heard that my colleagues contacted patients online in closed wards after clinical training was over. (Team 12)

### Type and distribution of complex dilemmas

**Fig. 2:**
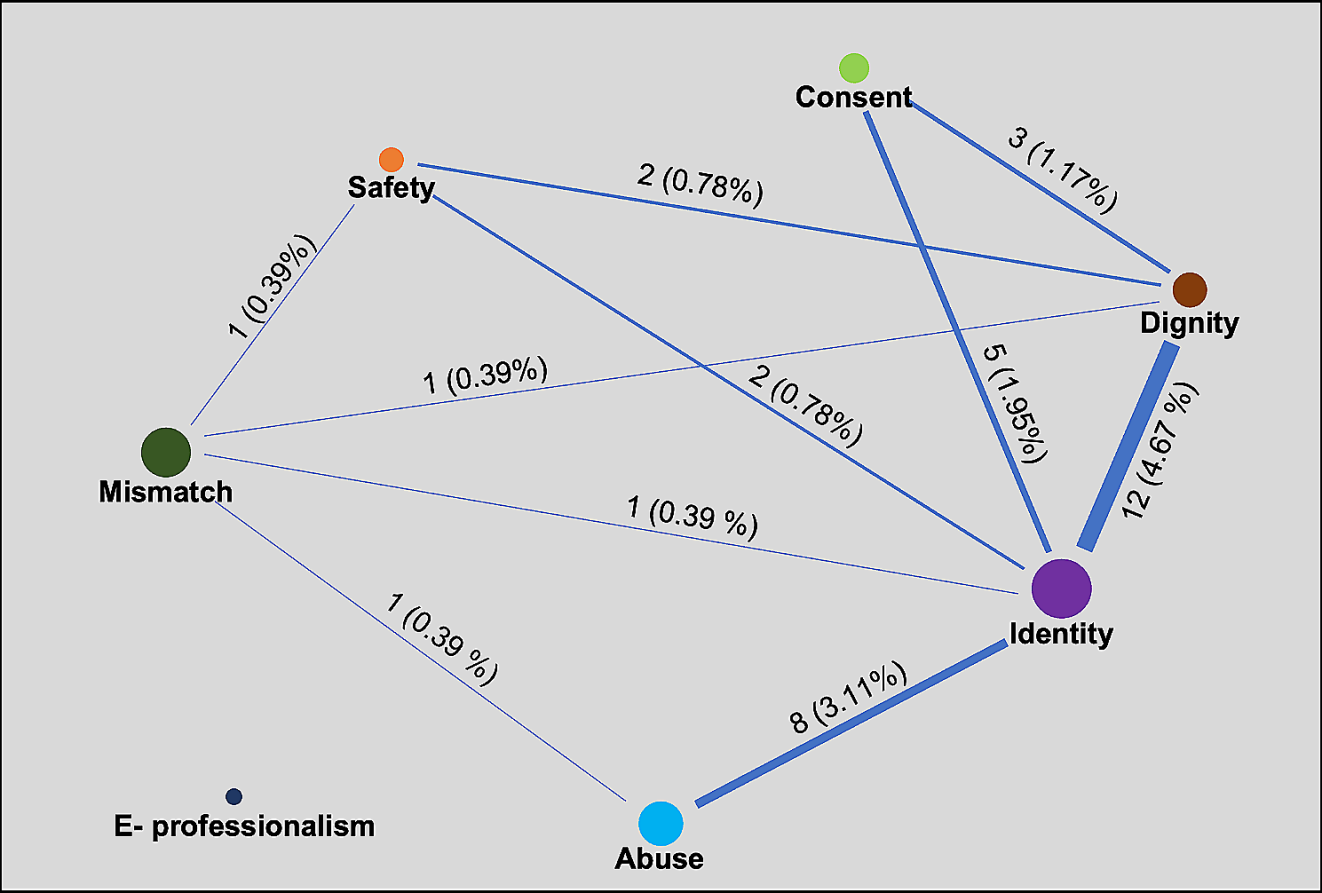
Visualized map showing the relationship between each dilemma theme. The line width reflects the relationship between the two themes, and thickness shows the distribution of frequency. The size of the circle representing each theme shows the number of single dilemmas. The number and percentage represent the number of complex dilemmas that consist of themes at both ends of a line and the distribution of the complex dilemmas. There were no narratives of e-professionalism corresponding to the complexity dilemma

Among the total of 257 dilemma narratives analyzed, it was discovered that 36 narratives involve more than one dilemma theme. In other words, situations with two or more dilemma contexts were classified as “complex dilemmas” (see Table 1). The types and distribution of “complex dilemmas” is shown in Fig. 2. The different models of MAXMaps (MAXQDA visual tools) can organize and explore data as well as visualize complex relationships. Figure 2 was composed using the data that were used to count the number of times two or more codes were assigned to the same narratives. The figure enables researchers to visualize the co-occurrence of codes as a network structure.

The researchers describe the three dilemma themes that account for the highest frequency in the results and add the frequency and narrative examples of the remaining dilemmas to the “Supplementary Material 1.” The top three (3) complex dilemmas were: “identity-dignity,” “identity-abuse,” and “identity-consent.”

The highest frequency among the complex dilemmas was for that of “identity-dignity” (*n* = 12, 4.67%). This dilemma implied that when students violated the dignity of patients in the clinical field, it was due to their status of being student-doctors. The identity-dignity-related dilemma was, representatively, about “the patient’s right to know.” An example of this is, a deathbed patient might ask about the nature of their disease or how long more they will live, which would put the student in a dilemma regarding whether they should attempt answering these questions as mere student-doctors, or insist on not telling the truth to the patient in accordance with the instructions of the professor and/or supervisor.

The second ranked dilemma was the “identity-abuse-related dilemma” with a frequency of *n* = 8 (3.11%). This dilemma was often related to the students’ status as student-doctors, where they were “deprived of legitimate learning opportunities” or “discriminated” in the clinical workplace. An example of this is in relation to medical records, where clinical education was restricted because the student did not have the right to access medical records or the information that could be read was limited. Additionally, students often felt tired of “walking on eggshells (Nun-chi)” around professors and nurses. They had the right to clerkship education; however, they experienced the feeling of being outcasts. The “identity-consent” was also a major dilemma faced by medical students (*n* = 5, 1.95%). This dilemma occurred when informing patients that it was a clinical education situation or when receiving consent for physical examination as a result of the student’s status as a student-doctor.

## Discussions

This study analyzed the contents of group-discussions on the experiences with regard to students’ dilemma in order to explore the dilemmas encountered by students during clerkship. The contents of the dilemma discussions were examined in comparison with the professional dilemmas framework proposed by Monrouxe and Rees [20], and the following major discussion points were derived.

First, the most common dilemma theme was the “identity-related dilemma.” The analysis of students’ discussion narratives revealed that the distribution of single dilemmas was in the following order: “identity,” “abuse,” “mismatch,” “dignity,” “consent,” “safety,” and “E-professionalism-related dilemma.” Among them, 35.8% were “identity-related dilemmas.” Additionally, even in the case of “complex dilemmas” where two dilemma themes appeared in a single narrative, “identity”-related themes were combined with the themes of “dignity,” “abuse,” and “consent,” therefore showing a high frequency. The “identity-related dilemma” that repeatedly appeared in this study was due to the status of medical students as “student-doctors.” As a result of students being denied participation by professors, nurses, and patients during their clerkship, they voluntarily or involuntarily do not identify themselves as students.

Previous studies have reported several countries dealing with identity-related dilemma, and these studies have mainly dealt with the low status of students and limited roles during clerkship [34]. A study reported that medical students often felt like “spare wheels” or “unskilled, anonymous members” of a large group of clinicians [4]. They were recognized as “only observers” because of their inability to perform treatment independently, and their duties as well as their roles as student-doctors were vague [35]. The clinical workplace of healthcare workers represents a hierarchical relationship structure, and medical students easily find themselves in a dilemma as a result of occupying the lowest position in the hierarchy [34]. Students easily feel inferior in such situations, and even when faced with a situation that goes against their conscience and values, they may passively avoid it rather than actively respond [9]. Previous studies have reported that the period of clinical clerkship, which should function as an educational field, limits students’ legal participation due to the identity problem of “student-doctor.” This study reaffirms this fact. It is well known that positive student experiences in the clinical environment contribute to professional identity formation. However, considering that complete control over the real world of medical practice is difficult to achieve, reflection upon the negative dilemmas that students experience and educational support to guide them in learning are essential [36].

Second, the researchers’ findings showed newly derived dilemma themes from the dilemmas proposed by Monrouxe and Rees [20]. “Mismatch” was among the top three dilemma situations in our study. It refers to the gap between “what they knew,” that is, the knowledge they learned in the classroom and “the actual practice at the workplace.” The students also noted that they felt a difference in the way they worked in each specialty when they rotated among multiple specialties.

Previous studies have reported that medical students face difficulty in applying classroom theories to practical clinical settings [15, 37–39]. Improving the manner in which lecture-based knowledge is “transferred” and “integrated” with the practical clinical knowledge is an important goal in medical education [39, 40]. It is important to note that these difficulties put medical students in such a dilemma that they often fail to support either side between the knowledge obtained during studies and the knowledge obtained through practical experience. These problems can be interpreted based on the context of the clinical clerkship curriculum and teaching hospitals associated to Korean medical schools. In South Korea, teaching hospitals emphasize not only the role of educational institutions in nurturing medical students but also the function of tertiary general hospitals with regard to the treatment of severe diseases. The modernization of medical care in Korea began in the 1980s, and various social and medical security systems have been established in the country. As a result, the number of hospitals in Korea has increased significantly to improve the accessibility of hospitals for people. In Korea’s market economy, it was not natural for hospitals to focus on patient-care to remain viable. Therefore, professors at educational hospitals had no choice but to devote more time to patient care, and their professional evaluation is based largely on “treatment results” and “research performance” as opposed to “student education”; thus, the time to participate in student education is relatively reduced [41]. The “identity-related dilemma,” which had a high frequency in the research results, is also relevant in this context. When medical students’ education is relegated in the institution’s priority list, the dilemma situation experienced by students is bound to intensify.

In the sub-theme, “Nun-chi” is also a dilemma situation that has emerged in the South Korean medical context. “Nun-chi” is defined as the ability to evaluate social situations and understand others’ intentions and emotions through implicit cues. Koreans often use “Nun-chi” in social situations, and it is usually expected and desirable to have “Nun-chi” among Koreans when interacting with others [42]. In the Korean clinical workplace, “Nun-chi” is considered an essential skill for effective clinical practice as it helps to grasp the nuances of a situation and facilitates appropriate behavior in the communication process [43].

East Asian countries, including South Korea, are collectivist or high-context societies—where they assign considerable value to one’s relationship with their group and with others. The concept of “Nun-chi” is also closely related to collectivism and high-context communication, which has been greatly influenced by Confucianism in East Asian countries. In such countries, the concept of the self is formed in connection with others in the sociocultural context of the country as opposed to focusing on individuality and independence [44]. Thailand’s “Kreng Jai” is a similar concept and emphasizes the consideration for others and the need for self-suppression [45]. The students in Confucian countries, such as Indonesia, Thailand, and China tend to exhibit “high levels of loyalty and respect for their seniors and have special relationships with school alumni during their clerkship” [9, 14].

However, according to previous studies, “Nun-chi” should not be interpreted as a product of a collectivist culture that contrasts with individualism. It can be a product of passive concession and consideration to care about the feelings of others. However, sometimes it can be an active expression of emotion chosen to take care of individual interests in conflicting with others [46]. This argument can be explained by referring to the cultural context and how Korean medical students become intellectually mature over a period of time. In addition, Korean medical students display overall academic excellence as they face tremendous competition [47]. To further enhance their capabilities, it is necessary to facilitate their transition from a lecture-based learning environment to a realistic and practical learning environment, which requires, and develops, new competencies [48].

For students who have to undergo clerkship in the highly competitive environment of Korean medical schools, “having Nun-chi” is likely to have emerged as a social competency for interpersonal relationships, which later developed into a subtler skill during the clerkship period. Above all, given a situation where a professor who was a teacher at a medical school becomes a senior doctor after graduation, “making a good impression” is recognized as a major career task in addition to receiving good grades during their clerkship. Ginsburg and Lingard [49] show that students strategically use “impression management” to hide honest expression from their professors or senior doctors. In other words, Nun-chi can occur to avoid losses and secure gains with regard to interpersonal conflicts as difficult situations are avoided. The results of this study clearly revealed that students to intentionally asked questions despite knowing the answers, in order to portray a good image to the professor, of being “conscious of the resident’s mood” during the practice process.

This study had several implications. First, the pre-clerkship curriculum should make students aware of the realistic and practical clinical context and teach them how to overcome dilemmas, which can occur frequently in a complex and unpredictable clinical workplace. The students will always encounter dilemmas as they are inevitable; however, an important fact to note is that the dilemmas cause the students to suffer psychological and physical discomfort which hinders their ability to learn [20]. There is need for students to understand that the clinical field is a reality that is extremely complex and contains contradictory elements. It also simultaneously offers the opportunity to acquire relevant knowledge and information [49].

Additionally, students must be informed in advance about any discrepancy between the classroom theory and the practical workplace. This can be done by constructing a curriculum that allows both students and supervisors to discuss the dilemmas they may face in the workplace. In this case, group discussions contain real narratives that vividly reflect stories from the clinical workplace. These narratives can be integrated with knowledge gained from both formal and informal curricula [50, 51]. On the other hand, role modeling can be an alternative to this curriculum. Physicians are not merely ‘playing a role’ as actors might; instead, they are ‘embodying’ different types of roles. The cognitive and behavioral processes associated with successfully internalizing roles, such as that of a good doctor or medical educator, are crucial. In the real practice of clinical clerkship, students may encounter both positive role models and negative role models, potentially leading to dilemmas. Therefore, it is important to assist learners in developing strategies for identifying both good and poor role modeling, as well as employing conscious reflective maneuvers to cope with the potential influence of these models. Learners should also be supported in creating safe spaces for reflecting on negative role modeling and transforming it into effective learning experiences [36].

Second, there’s a necessity to develop educational content that addresses both local cultural and social needs. Medical graduates are adapting to new working methods, and the demand for training in foreign work environments is on the rise. Thus, it’s crucial to nurture global citizens who can comprehend the socio-cultural contexts of different countries [52, 53]. Additionally, it’s essential to instill reflexivity in them, enabling them to embrace cultural differences [53]. Every country has its own unique cultural traits and behaviors; thus, failing to understand these nuances and differences can be deemed unprofessional [54]. In addition to being aware of the prevailing Western-centered principles of professionalism, education addressing specific dilemmas that align with the distinct cultural characteristics of various countries is necessary [55].

This study has several limitations. Firstly, as it was conducted in Korean medical schools, the findings might not fully encapsulate the clinical contexts of medical schools in other countries. Clinical educational environments differ between Eastern and Western settings, especially in terms of recognizing context-specific and culture-specific concepts like medical professionalism [56]. For instance, even within the same medical school, clinical clerkship experiences can vary based on the size and location of the hospital. Secondly, this study relied on student group-discussion data, which limited the depth of analysis. Unlike individual interviews, the researchers couldn’t ask follow-up questions to delve deeper into students’ narratives. Nonetheless, students were candid in their discussions, knowing their responses would remain anonymous in the study. Thirdly, the research employed the template analysis method. While effective, this method has certain constraints related to the openness of data due to the structured coding process inherent in qualitative research. However, its flexible application in this study led to the emergence of new themes. Moreover, the parallel coding capability of template analysis allowed the researchers to categorize a single narrative under multiple codes, highlighting complex dilemmas. Finally, it is necessary to consider the effect of the COVID-19 pandemic on the curriculum. This study was conducted as a qualitative study; however, with COVID-related conditions in hospitals, this effect could not be strictly controlled. Nevertheless, the dilemmas experienced by students seem akin to those encountered before the COVID-19 pandemic. In South Korea, while most courses transitioned to online classes, clinical clerkships largely continued in hospitals, except during the most severe COVID-19 situations [47].

## Conclusions

This study delved into dilemmas medical students face during clinical clerkships, with the predominant issue relating to their identity as student-doctors. This challenge persisted amidst varied, complex situations. The results underscore difficulties due to the students’ roles in clinics. Clinical settings, while educational, often adopt informal curricula, resulting in inconsistent instruction. Notably, compared to earlier dilemma frameworks, this study spotlights the deep influence of national contexts on such dilemmas. In Korea, while educational hospitals are vital learning platforms, the pressure for premier medical services in major hospitals can impede stable student education. The term “Nun-chi” used by Korean students suggests proactive strategies to address dilemmas related to social aptitude, a valued Korean trait. Overall, this research underscores dilemmas tailored to South Korea’s unique socio-cultural environment, offering key insights to refine medical training and understanding of professional dilemmas.

### Electronic supplementary material

Below is the link to the electronic supplementary material.

Table S1. Students’ narratives of complex dilemma

## Data Availability

The datasets of this article are available from the corresponding author on reasonable request.

## Abbreviations

PDS 3−1: Patient-Doctor-Society 3−1
HYUCM: Hanyang University College of Medicine
