# Supplementary material for: The hidden hurdles of clinical clerkship: unraveling the types and distribution of professionalism dilemmas among South Korean medical students
Source: BMC Med Educ. 2024 Feb 15;24:150. doi: 10.1186/s12909-024-05115-9 (PMC10870601; doi:10.1186/s12909-024-05115-9)
Supplement: Supplementary file 1 — Table S1. Students’ narratives of complex dilemma [file 12909_2024_5115_MOESM1_ESM.docx]

# Supplementary Information

**Appendix. Students’ narratives of complex dilemma**

| Types of complex dilemma | N (%) | Students’ Narrative |
| --- | --- | --- |
| Identity + Dignity | 12(4.67) | I learned to say, “I’m student-doctor OO,” but in reality, patients don’t prefer a student to be in charge. So, in certain departments, we were instructed to say, “I’m just training,” and not reveal that we were student-doctors. I felt guilty **of using the patient as a “tool for presenting my case.**” (Team 1) |
| Identity + Abuse | 8(3.11) | The conditions for receiving data on patients were limited. It was difficult to determine **whether to use an Electronic Health Record (EHR). All information was not accessible because I was not a doctor, just a student-doctor**. (Team 15) |
| Identity + Consent | 5(1.95) | I said to a patient, “Hello, I'm OOO, a student-doctor. I have a question; do you have time?” The patient said, “If you are a doctor, you are a doctor, and if you are a student, you are a student—what is a student-doctor? No, I don’t want to answer.” (Team 23)  When I informed the resident about this, he changed the case and said: **“I think you don’t need to introduce yourself as a student-doctor.”** I thought whether it was a priority when caught between “patient consent” and “smooth clerkship practice.” (Team 28) |
| Consent + Dignity | 3(1.17) | In the obstetrics and gynecology department, **the student-doctor participated in the clinical practice without the patient’s consent** when the patient was in a lithotomy position and blindfolded. (Team 4) |
| Identity + Safety | 2(0.78) | During surgical practice, my resident trainer provided me an opportunity to suture after laparoscopic surgery. As I was still not good at it, the trainer asked me to “tie” and “untie” again and again. I repeated this process several times, and **I was worried that it might damage the patient’s skin.** (Team 11) |
| Safety + Dignity | 2(0.78) | **The resident administered the wrong dose of antibiotics**. The nursing department advised me not to tell the patient about this, and the professor managed the situation by instructing the erroneous resident to look for reference cases related to antibiotic doses. (Team 8) |
| Mismatch + Identity | 1(0.39) | A professor advised me that during interviews and physical examinations, I should say that I am a doctor because certain patients don’t like student-doctors. Another professor advised that I must say that I am a student-doctor. Different specialties had different guidelines. (Team 13) |
| Mismatch + Safety | 1(0.39) | Drug side-effects need to be regularly checked after prescribing drugs. It was necessary to check the side-effects of psychiatric drugs every week, but the outpatient clinic was pushed back a month and it was difficult to check the side-effects. (Team 29) |
| Mismatch + Dignity | 1(0.39) | **I’ve learned to listen intently** when treating a patient. However, when a patient complains of non-specific symptoms or simple abdominal pain**, medical staff may become disinterested**. After a patient left, the staff remarked, “He looks like an NP (neurosis patient), right?” Sometimes I too was bored with a patient and thought: “I wish the treatment would be over soon.” (Team 23) |
| Mismatch + Abuse | 1(0.39) | The resident suggested the prescription should be NS (normal saline) but the professor persisted with 5DS (dextrose water). To my surprise, the professor even scolded the resident for this, and I learned that a professor’s experience is more important than textbook information.(Team 13) |
| Total | 36(14.01) |  |
